# Concomitant Idiopathic Intracranial Hypertension, Normal Pressure Hydrocephalus, and Pleomorphic Xanthoastrocytoma: A Case Report and Review of the Literature

**DOI:** 10.1155/2020/2420671

**Published:** 2020-09-02

**Authors:** Modhi Alhussinan, Turki Elarjani, Mohammed Jawhari, Mohammed Albrahim, Faisal Farrash

**Affiliations:** ^1^College of Medicine, Alfaisal University, Riyadh 11533, Saudi Arabia; ^2^Division of Neurological Surgery, Neurosciences Department, King Faisal Specialist Hospital and Research Centre, Riyadh 11211, Saudi Arabia; ^3^Division of Neurological Surgery, Neurosciences Department, King Fahad Medical City, Riyadh 12231, Saudi Arabia

## Abstract

**Background:**

Idiopathic intracranial hypertension (IIH) and normal pressure hydrocephalus (NPH) are disorders of the cerebrospinal fluid (CSF) flow dynamics. Pleomorphic xanthoastrocytoma (PXA) is a rare low-grade astrocytoma (World Health Organization grade II) representing <1% of astrocytomas. Combination of IIH and NPH with PXA is unheard of, with few published cases discussing the association of CNS tumors with either IIH or NPH, but never combined. We present a case of a 51-year-old woman with such a rare combination. *Case Presentation*. A fifty-one-year-old obese female presented with a progressive visual loss, abducens nerve palsy, and headache for three months. Further investigations revealed a right frontal tumor and ventriculomegaly on magnetic resonance imaging. Her symptoms improved remarkably after total surgical excision of the tumor with a ventriculoperitoneal (VP) shunt.

**Conclusion:**

The pathophysiology behind NPH and IIH is still not fully understood, yet their management is mostly dependent on CSF diversion. The concomitant development of two different CSF dynamic diseases with a PXA has not been reported in the literature. We hypothesize that PXA may have sparked an abnormal CSF circulation pattern and ventriculomegaly.

## 1. Introduction

IIH, widely known as pseudotumor cerebri, and NPH are disorders attributed to the CSF circulation dynamic. Although they have different pathophysiologies and opposite diagnostic features, they are primarily managed by CSF diversion [1]. NPH frequently develops in adulthood and presents with a triad of dementia, gait imbalance, and urinary incontinence [2]. Conversely, IIH is a syndrome with a higher tendency to present in obese young women. It is characterized by an abnormal elevation of intracranial pressure without a known cause [3]. Symptoms range between headaches, transient visual loss, pulsatile tinnitus, and ocular pain [4, 5]. Both IIH and NPH usually lack an association with space-occupying lesions [4]. We report a rare case of a patient presenting with signs and symptoms of IIH and NPH, with an incidental finding of a right frontal PXA. To our knowledge, no such case is mentioned in the literature.

## 2. Case Description

**Figure 1:**
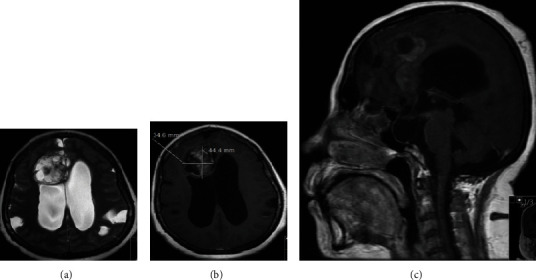
Axial view of a T2-weighted image with a right frontal intra-axial lesion measuring 34 × 44 mm in the transverse and anteroposterior dimensions, respectively. The lesion indents the ependymal surface of the right lateral ventricle. The lesion is heterogeneous with cystic loculations indicating necrosis; an associated bilateral ventriculomegaly of the lateral horns is appreciated (a). The lesion has a heterogeneous enhancement pattern in a T1-weighted image with contrast (b). T1 with contrast sagittal view revealing a scalloped corpus callosum with empty sella (c).

**Figure 2:**
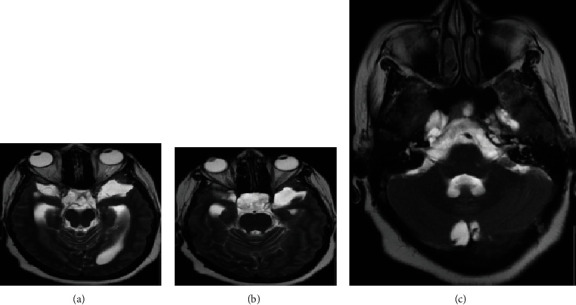
Axial view of a T2-weighted image of the midbrain (a) and upper pons lower midbrain (b) levels showing expanded temporal horns, increase in the subarachnoid space of the optic nerves, optic nerves' tortuosity, and deviation of the optic chiasm towards the left side, all indicating high ICP. The middle pons level shows bilateral Meckel's cave expansion and scalloping of the right petrous apex associated with a cephalocele (c).

A fifty-one-year-old obese lady with a body mass index of 45.3, known to have hypertension and dyslipidemia, was referred to our institute as a case of a right frontal mass lesion associated with hydrocephalus (Figure 1). Her symptoms started three years ago when she gradually developed gait imbalance with frequent falling, urinary incontinence, headache, dizziness, confusion, and personality changes. Two years later, she had a progressive bilateral visual acuity deterioration that initially started as photophobia, progressing to blurred vision, then to hand motion perception only. (Figure 2).

**Figure 3:**
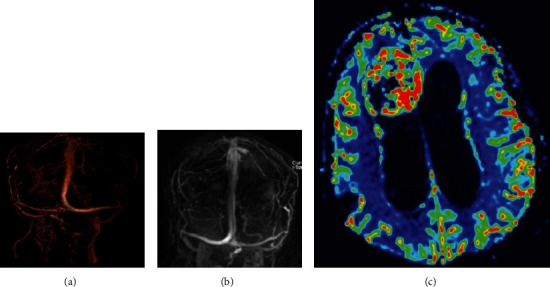
MRV of the cerebrum that shows patent superficial and deep venous system (a, b). MR perfusion shows the high perfusion hinting towards a high-grade nature of the mass (c).

Physical examination showed a fully awake patient with a nonreactive right pupil of 5 millimeters and a sluggish left pupil of 6 millimeters in size; ophthalmoscope examination showed bilateral pale edematous optic disk, right oculomotor cranial nerve palsy, left abducens cranial nerve palsy, and a decreased sensation of the entire right side of the face to pinprick. Upper and lower limb sensory and motor examinations were normal. The patient was investigated with an MRI, magnetic resonance venography, and magnetic resonance perfusion that showed patent venous sinuses (Figure 3). The MRI showed a large right frontal periventricular heterogeneous enhancing frontal tumor with a ventriculomegaly and dilated CSF spaces.

The patient underwent a right frontal craniotomy for tumor resection and insertion of an external ventricular drain as her intracranial pressure (ICP) was very high intraoperatively ranging between 28 and 30 cmH_2_O; initially, the drained CSF was xanthochromic then started to clear gradually. The physical exam postoperatively shows normal movement of all her limbs with reactive bilateral pupils. Histopathological assessment was evident for large areas of necrosis without a nuclear pseudopalisading or significant mitotic activity, which is consistent with the features of grade II WHO PXA.

**Figure 4:**
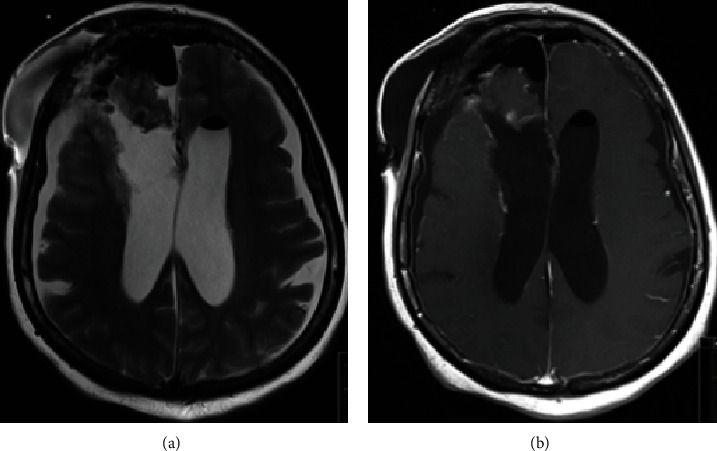
Postoperative axial view of a T2 and T1 with contrast-weighted images, showing total resection of the tumor (a, b).

Serial postoperative examinations showed remarkable improvement in her presenting symptoms, including visual acuity, gait, and urinary incontinence. Postoperative MRI showed complete resection of the tumor (Figure 4). The patient underwent a right VP shunt insertion and EVD removal two weeks after her initial surgery and was discharged in a better condition. Her outpatient exam three-month postoperation revealed no abnormal gait disturbance or visual deficits.

## 3. Discussion

To our knowledge, no similar case of a simultaneous IIH, NPH, and PXA was mentioned in the literature. However, there have been a few cases that reported associations of NPH or IIH with a space-occupying lesion (Table 1). There was one case series of two patients who presented with symptomatic NPH as an unusual presentation of a supratentorial extra ventricular space-occupying lesion [4]. The first patient was a fifty-eight-year-old female who had marked dementia associated with gait difficulties and urinary incontinence. Further investigation revealed a meningioma in the left temporoparietal region. One month after total resection of the meningioma, follow-up examination revealed a significant improvement of the gait abnormalities and incontinence with decreased ventricular size.

The second case was a sixty-eight-year-old male patient who presented with involuntary left arm movement, followed by progressive gait impairment and urinary incontinence. Neuroimaging studies revealed a lesion in the right frontotemporal region. Partial resection was done, and the histopathological diagnosis demonstrated a glioblastoma (WHO grade IV). However, due to the tumor progression, the patient died after three months [4]. Another case series has described three patients with the diagnosis of meningioma associated with an IIH. The first patient had an abducens nerve palsy, the second and third patients both had a decreased visual acuity associated with a visual field defect. All three patients have experienced marked symptomatic improvement after CSF diversion [6].

Another case report has described a 27-year-old woman who presented with a three-day history of headache and progressive visual loss in both eyes. Initially, she received a diagnosis of IIH as the computed tomography scan revealed no abnormal findings, and a lumbar puncture yielded a CSF pressure of 430 mm of H_2_O. The patient was started on acetazolamide and dexamethasone; however, she did not show any improvement. MRI was conducted one week after admission and revealed a discrete signal hyperintensity of the gyri surrounding the left central sulcus with no mass effect. Five weeks later, a VP shunt was placed due to visual deterioration, and CSF examination revealed glial fibrillary acidic protein-positive cells with no anaplasia. Brain biopsy showed the characteristic features of a PXA [7].

The case we described combines three different and unrelated disease entities, with an independent pathophysiological mechanism. As in our patient, the presentation of the case by Delgado-Alvarado et al. [7] generates a question: could PXA induce CSF structural or dynamic changes that might have triggered one or two of the accompanying pathologies? The Monro-Kellie doctrine states the equilibrium between the brain, CSF, and intracranial blood volumes. Any changes in one of the volumes would lead to an opposite effect of the other volumes. An example would be a brain edema following a head injury would expand the brain volume and reduces the CSF and blood volume [8]. Therefore, we speculate that PXA may have altered the CSF flow dynamics, leading to ventriculomegaly with NPH clinical presentation and a defect of CSF absorption that leads to an elevated ICP and an IIH clinical presentation.

## 4. Conclusion

We report the first case of a simultaneous IIH, NPH, and PXA. No clear association can be described between them; however, it may be related to PXA-induced ventriculomegaly with NPH clinical presentation and a defect of CSF absorption, leading to IIH clinical presentation.

## Figures and Tables

**Table 1:** Summary of similar cases in the literature.

Reference	Age/sex	Clinical presentation	Type and location of the tumor	IIH, NPH, or hydrocephalus	Management	Outcome
Naydenov et al. [6]	58/F	Six-month history of progressive gait disturbances, urinary incontinence, and dementia	Left temporoparietal meningioma	NPH	Total resection	Transient right-hand paresis that resolved after 1 month. Symptoms of NPH improved after 1 month
Naydenov et al. [6]	68/M	Eighteen-month history of involuntary LT arm movement + progressive gait disturbances and urinary incontinence	Right frontotemporal glioblastoma	NPH	Partial resection	Died 3 months later from the consequences of glioblastoma
Delgado-Alvarado et al.[7]	27/M	Progressive vision loss px: papilledema	Pleomorphic xanthoastrocytoma	IIH	VP shunt	Not mentioned
Sharma et al. [4]	32/F	Refractory headache + right 6^th^ nerve palsy	Multiple meningiomas	IIH	Resection of the largest lesion followed by VP shunt 7 months later	Complete symptom resolution 6 weeks after VP shunt insertion
Sharma et al. [4]	40/F	Nausea, tinnitus, headache, visual defect (central and inferior visual field defect in her left eye), and papilledema	Meningioma	IIH	Left VP shunt for IIH management followed by meningioma resection	Significant visual improvement; other symptoms persisted
Sharma et al. [4]	49/F	Left visual field deficits and papilledema	Parietooccipital meningioma	IIH	Right frontal VP shunt followed by gamma knife radiosurgery 1 year later for the meningioma	Significant symptom improvement and resolution of visual complaints
Present case	51/F	Gait imbalance, urinary incontinence, confusion, headache, and progressive visual loss	Frontal PXA	NPH and IIH	Surgical resection and VP shunt	General symptomatic improvement

## Abbreviations

MRI: Magnetic resonance imaging
MRV: Magnetic resonance venography
ICU: Intensive care unit
NPH: Normal pressure hydrocephalus
IIH: Idiopathic intracranial hypertension
PXA: Pleomorphic xanthoastrocytoma
ICP: Intracranial pressure
CSF: Cerebrospinal fluid
WHO: World Health Organization
VP shunt: Ventriculoperitoneal shunt.
